# Clinical Observations in Private Practice

**Published:** 1851-09

**Authors:** E. B. Haskins

**Affiliations:** Clarksville, Tenn.


### WESTERN JOURNAL

OF

### MEDICINE AND SURGERY.

SEPTEMBER, 1851.

ART. I.—*Clinical Observations in Private Practice.* By E. B. HASKINS, M.D., of Clarksville, Tenn.

(CONTINUED FROM MARCH NUMBER, PAGE 191.)

OBSERVATION IV.—*Three Cases of Simple Spermatorrhea.*

CASE I.—In the spring of 1846, I was consulted by Mr. ——, a young gentleman, aged about 25 years, of rather stout frame and of a good constitution. He had been the subject of gonorrhea about twelve months before, but was relieved in a short time, and had no return of the disease. He had been guilty, as he declared, of great venereal excesses; that he had attempted to reform his habits on account of a "nervous weakness" he thought his excesses were bringing upon him; but instead of the relief he expected, he was growing worse from nightly emissions of semen—occurring every night, and sometimes more than once.

Bowels regular; appetite moderately good; is restless at night; easily fatigued; desponding in spirits; urine, when left to stand a short time, exhibits the ammonio-magnesian pellicle; otherwise healthy; no hemorrhoid, or other sources of irritation about the rectum.

℞. Pulv. camph., } ää ℈ij.

Extr. hyosci., }

Sulph. quin., } ää ℈j.

Sulph. ferri, }

M. ft. Pil., No. xl.

One pill to be taken at a time, morning and night, for two weeks, then omit for a few days, and again resume them; exercise temperately on foot; sleep on a hard bed with light covering; and refrain from indulging erotic ideas.

Health was restored in about eight weeks.

Case II.—July, 1847, Mr. ——, aged about 24 years, low in stature, and muscular; rather pale complexion, and of good moral habits; informs me that he has in prospect a matrimonial connexion, but thinks he is not in a condition to fulfil his engagements.

He says he is greatly troubled with nocturnal emissions, and, indeed, is so very excitable that he can, by the force of the imagination alone, provoke an emission at any time.

He feels no injury to his general health, except "a nervousness." Appetite good; sleeps well; bowels regular; no source of irritation in the anal region; has never had gonorrhea, or been addicted to sexual indulgance. Masturbation was not acknowledged, but was suspected from his manners, etc. Urine reported to be healthy.

Treatment was the same as in case 1st, except the addition of inunction of camphorated mercurial ointment over the perineal surface.

I heard no more from this case until the latter part of October, when he had been married about three weeks.

He informed me that he had used no other remedies than those ordered by me, and that he had regained his health prior to the consummation of his engagement.

CASE III.—September 1, 1849, Mr. ——, aged about 26 years, of bilio-nervous temperament, small frame of body, but good constitution, complains to me of debilitating nocturnal emissions.

He has been subject to nocturnal seminal emissions for the last two years. They at first recurred at distant intervals, but they have gradually grown more frequent, until he now is disturbed by them nearly every night, and he feels that his health is beginning to suffer from them.

He complains of occasional palpitation of the heart; dull headache, languor, and indisposition to mental or bodily exertions; rest at night often disturbed by "bad dreams;" appetite failing; bowels constipated; urine abundant, pale, slightly acid, and of low specific gravity; no hemorrhoids or anal fissures.

He is chaste in his habits, but acknowledges that he practiced onanism freely in the early part of his life; has never had gonorrhea.

Treatment pursued was the same as in case 1st, with the addition of pills of compound extract of colocynth to keep the bowels open.

Nothing novel is claimed for the cases here recorded. They constitute what Lalemand would term the "most simple cases of nocturnal pollutions," and for which he would prescribe the artificial sulphuretted baths. According to Dr. Dunglison, however, such cases of nocturnal pollution as do not "occur more than once in the course of the night, and every night, ought scarcely, in a young and robust individual, to be esteemed a pathological condition."* Without stopping to inquire wheth-

*Cyc. of Prac. Med. (Amer. Ed.) Art. Spermatorrhea.

er or not such activity of the seminal secretion, or irritability of the vesiculæ seminales, has overstepped the physiological bounds, it will hardly be questioned that every individual, however young and robust, who desires decent and comfortable repose, would be glad to be relieved of such annoyance. Besides, whenever any organ has reached the maximum of functional activity, the cautious practitioner will rather anticipate the pathological accession, by prudent steps to reduce the function to as near the medium of activity as is compatible with health.

On this point I would further remark, that I have never known robust health to be, *per se*, a cause of spermatorrhea. I have had many cases of the kind under my notice, and in the absence of all local sources of irritation, I have always found, or had good reason to suspect, that abuse of the sexual passion was the prime cause of the malady.

Observation V.—*A Case of Spermatorrhea; complete impotence; urethral irritation; cauterization; partial relief; facial palsy.*

July, 1848, I was consulted by Mr. ——, who is 21 years of age; six feet high; spare frame; stoops in the shoulders; large hands and feet; small head; restless and timid expression of countenance; but little beard. Genital organs above the medium size, and of healthy appearance, except a slight tumefaction at the end of the glans penis, which was smeared over with a gum-like secretion.

He gave the following history of himself: He is the only surviving offspring of wealthy parents; was born and reared in South Carolina. At about 12 years of age he commenced the practice of masturbation, which soon grew into a habit, that was continued until about eighteen months ago, when the state of impotence rendered abortive his efforts. From this time nocturnal pollutions (and perhaps diurnal also) harrassed him. In a short time, he lost all amative feeling. After the age of puberty he did not practice masturbation oftener than three times a week, on an average; is not positive, however, as to that. He was early impressed with the impropriety of his practice, and made frequent ineffectual attempts to reform; has been traveling for the last three years in Europe. He has never had sexual intercourse, and therefore has never had venereal disease. He has had no venereal desires for more than twelve months;—made several ineffectual efforts at venery before he lost all inclination, but failed on account of imperfect erection. He could at that time practice onanism with perfect and prolonged erection. He has now sometimes partial erections, but without desire, and they are painful; he is much troubled with emissions at night, sometimes during dreams, but not pleasureable. Most of the emissions are without any erection, and attended with no pleasure.

His general health is good. He is fond of hunting and fishing, and takes a good deal of exercise; nor is he easily fatigued. There are observable some muscular twitchings about the face and eyelids, particularly after emissions at night.

Urine natural in quantity, and of healthy appearance. For want of a microscope it was not examined for spermatozoa.

On passing a bougie down the urethra, a point of irritation was discovered in the prostatic portion of that canal.

The length of the canal being taken on a silver catheter, the point of irritation was cauterized with Lalemand's *porte caustique.* Sulphate of iron, as a tonic, was ordered twice a day, and a cold infusion of buchu to be daily drank; with injunctions to take regular but moderate exercise on foot, or in a carriage, sleep on a hard bed with little covering, and avoid lying upon the back; and to bathe the genital organs three or four times a d.iy with cold water. Diet to be nourishing.

By the first of September, there was evident improvement. The nocturnal emissions had not been so frequent, and the patient said he had some return of sexual desire, with slight and transient erections. Within the last two or three days, however, he had suffered some increase in the frequency of his emissions.

The urethra was again cauterized, (Sept. 3), and the original treatment renewed.

In a few days, my patient called at my office, carpet-bag in hand, saying that he was hurrying to a steamboat lying at the wharf, to take passage for Nashville, and thence home; that he was much better; had not had an emission since the last cauterization; but that he wished me to tell him what was the matter with his face. He was affected with facial palsy, with complete lagophthalmia of one side. The paralysis had come on him the night before, without his consciousness, and there was no feeling of indisposition at the time of the interview. As he was determined to go on, I explained to him the critical nature of his condition, placed a bandage over the affected eye, gave him a purgative, and advised him to place himself under medical treatment as soon as he reached Nashville. I learned, indirectly, that he did so, and after a long confinement he was restored to health. I have had no positive knowledge of his case since he left me. It should have been mentioned before, that the night before the attack, he slept upon the floor, exposed to a current of air.

*Remarks.*—This case loses much of its interest, for the want of a continued history.

It is by no means a new circumstance for the brain and nervous system to suffer injury, and that irreparably too, from an exhausting spermatic flux; yet each form of cerebral or nervous lesion, if carefully observed, and faithfully reported, may, in time, throw some light upon the remarkable pathological relations existing between the brain and nervous system and the spermatic organs.

*(To be continued.)*

